# Extending the scope of coiled-coil crystal structure solution by *AMPLE* through improved *ab initio* modelling

**DOI:** 10.1107/S2059798320000443

**Published:** 2020-02-25

**Authors:** Jens M. H. Thomas, Ronan M. Keegan, Daniel J. Rigden, Owen R. Davies

**Affiliations:** a Institute of Integrative Biology, University of Liverpool, Liverpool L69 7ZB, England; b Research Complex at Harwell, STFC Rutherford Appleton Laboratory, Didcot OX11 0FA, England; c Institute for Cell and Molecular Biosciences, Newcastle University, Framlington Place, Newcastle upon Tyne NE2 4HH, England

**Keywords:** *AMPLE*, coiled-coils, molecular replacement, phasing

## Abstract

The phase problem remains a major barrier to overcome in protein structure solution by X-ray crystallography. In recent years, new molecular-replacement approaches using *ab initio* models and ideal secondary-structure components have greatly contributed to the solution of novel structures in the absence of clear homologues in the PDB or experimental phasing information. This has been particularly successful for highly α-helical structures, and especially coiled-coils, in which the relatively rigid α-helices provide very useful molecular-replacement fragments. This has been seen within the program *AMPLE*, which uses clustered and truncated ensembles of numerous *ab initio* models in structure solution, and is already accomplished for α-helical and coiled-coil structures. Here, an expansion in the scope of coiled-coil structure solution by *AMPLE* is reported, which has been achieved through general improvements in the pipeline, the removal of tNCS correction in molecular replacement and two improved methods for *ab initio* modelling. Of the latter improvements, enforcing the modelling of elongated helices overcame the bias towards globular folds and provided a rapid method (equivalent to the time requirements of the existing modelling procedures in *AMPLE*) for enhanced solution. Further, the modelling of two-, three- and four-helical oligomeric coiled-coils, and the use of full/partial oligomers in molecular replacement, provided additional success in difficult and lower resolution cases. Together, these approaches have enabled the solution of a number of parallel/antiparallel dimeric, trimeric and tetrameric coiled-coils at resolutions as low as 3.3 Å, and have thus overcome previous limitations in *AMPLE* and provided a new functionality in coiled-coil structure solution at lower resolutions. These new approaches have been incorporated into a new release of *AMPLE* in which automated elongated monomer and oligomer modelling may be activated by selecting ‘coiled-coil’ mode.

## Abstract 2

The solution of coiled-coil crystal structures may be achieved by *AMPLE* through the use of ensembled *ab initio* models in molecular replacement. Improvements in *ab initio* modelling of elongated helices and oligomeric coiled-coils allow *AMPLE* to solve a greater number of coiled-coil structures and at lower resolution than previously achieved.

## 1. Introduction   

The coiled-coil is perhaps the best understood protein fold, and in its ideal form constitutes a highly geometric structure that has been defined computationally (Lupas & Gruber, 2005 ▸). A theoretical model of the coiled-coil was first postulated in 1952 by Francis Crick (Crick, 1952 ▸, 1953*a* ▸,*b* ▸), guided by the characteristic α-form X-ray diffraction patterns of natural fibres, including hair and wool, that were previously collected by William Astbury (Astbury & Street, 1931 ▸; Astbury & Woods, 1933 ▸). In this classic model, the coiled-coil was described as two or three parallel α-helices that twist around one another at a crossing angle of approximately 20°, such that their hydrophobic side chains become interlocked in a ‘knobs-into-holes’ pattern that repeats every seven amino acids in a ‘heptad repeat’. An isolated α-helix has 3.6 amino acids per turn, with a rise of 1.5 Å per amino acid, providing an α-helical pitch of 5.4 Å per turn. Within the classic coiled-coil model, the interhelical crossing angle provides a periodicity of seven amino acids across two turns, with a pitch along the coiled-coil axis of 5.1 Å per α-helical turn (Lupas & Gruber, 2005 ▸; Hartmann, 2017 ▸; Lupas *et al.*, 2017 ▸). However, whilst this classical model proved to be correct, subsequent experimental evidence demonstrated that it represents only part of a large family of highly divergent structures. These include larger oligomers, parallel and antiparallel orientations, a range of inter-helical crossing angles, left or right super-helical handed­ness, interruptions by skips, stammers and stutters, non-heptad periodicities, deviation from ‘knobs-into-holes’ packing and variations of these parameters along the coiled-coil length (Lupas & Gruber, 2005 ▸; Parry *et al.*, 2008 ▸; Moutevelis & Woolfson, 2009 ▸; Lupas *et al.*, 2017 ▸; Hartmann, 2017 ▸). Thus, the modern definition of a coiled-coil encompasses a highly diverse family of elongated α-helical structures that exhibit a wide range of geometries and topologies.

As structure begets function, the diverse nature of coiled-coil structures underlies their ubiquitous role in highly divergent cellular functions. These include roles as mechanically rigid fibres, such as in hair, extracellular matrices and cyto­skeletal networks, filamentous assemblies within flagella, pili and phage-coat proteins, molecular spacers that separate functional domains across large distances, molecular rulers for catalysis, mediators of oligomerization such as transcription factors and molecular motors, and in scaffolding large architectural assemblies (Lupas & Gruber, 2005 ▸; Truebestein & Leonard, 2016 ▸). These diverse functions explain the presence of coiled-coils within 10% of eukaryotic proteins (Liu & Rost, 2001 ▸), and underlie the importance of their structure elucidation. Further, the geometry of coiled-coils means that the most basic understanding of their structure, namely oligomer state and their parallel or antiparallel orientation, can dramatically transform our understanding of the topology of their wider biological assemblies, such as when they mediate head-to-head association between functional domains (Davies *et al.*, 2015 ▸; Forment *et al.*, 2015 ▸). Thus, structure solution of coiled-coil proteins is fundamental to our understanding of a wide range of cellular functions.

The coiled-coil is an inherently challenging target for crystallographic structure solution. The diverse range of coiled-coil geometries and topologies makes the accurate structure prediction of non-ideal coiled-coils extremely difficult, thereby limiting our ability to identify suitable search models for molecular replacement. Additionally, small local perturbations in coiled-coil parameters can lead to substantial deviation of the super-helical axis along its length, which can be affected by crystal packing. Thus, even when the structure of the same protein or a close homologue is known, subtle long-range alterations in different crystal settings make coiled-coils typically poor candidates as search models in molecular replacement (MR). Further, the challenge of coiled-coil structure solution extends beyond structural diversity into common characteristics and pathologies of coiled-coil data sets, which we have observed in previous (Syrjanen *et al.*, 2014 ▸; Davies *et al.*, 2015 ▸; Dunce *et al.*, 2018 ▸) and ongoing studies, and which are in agreement with reports from other laboratories (Guzenko *et al.*, 2017 ▸; Blocquel *et al.*, 2014 ▸; Dauter, 2015 ▸; Caballero *et al.*, 2018 ▸). These include anisotropic diffraction, apparent translational noncrystallographic symmetry (tNCS), internal symmetry within coiled-coils, densely packed protein with low solvent content and the formation of recursive fibrous structures within the crystal lattice. These features hamper molecular replacement as there is often little difference between the agreement of correctly or incorrectly placed molecules with experimental data, and hinder experimental phasing as native intramolecular features often mask signals within anomalous difference Patterson maps, and crystals frequently exhibit poor reproducibility and non-isomorphism. These difficulties are likely to underlie the under-representation of coiled-coil structures within the Protein Data Bank that was reported by Peng *et al.* (2004 ▸).

Alongside the difficulties outlined above, the high α-helical content of coiled-coils offers a unique advantage to crystallographic structure solution owing to the rather rigid and well defined nature of helical fragments and the characteristic appearance of helical structure in electron-density maps. This has been exploited by a number of unconventional molecular-replacement methods that aim to solve coiled-coil structures in the absence of homologous structures or experimental phasing information, such as *AMPLE* (Bibby *et al.*, 2012 ▸; Thomas *et al.*, 2015 ▸). *AMPLE* utilizes *ab initio* structural models to provide potential search templates for molecular replacement and exploits the principle that common structural regions within a large number of *ab initio* models predict their local accuracy. Computationally cheap *ab initio* modelling, such as in *Rosetta*, is used to generate 1000 models, which are processed into ten aligned clusters of up to 30 polyalanine decoys, truncated at 20 levels, and subclustered at two radius thresholds, thus generating up to 400 ensembles of a wide range of sizes for MR (Bibby *et al.*, 2012 ▸). These ensembles are fed into the *MrBUMP* pipeline (Keegan & Winn, 2007 ▸, 2008 ▸; Keegan *et al.*, 2018 ▸), which runs *Phaser* (McCoy *et al.*, 2007 ▸) for each ensemble, followed by density modification and main-chain tracing using *SHELXE* (Sheldrick, 2015 ▸; Caballero *et al.*, 2018 ▸), and optional subsequent model building by *ARP*/*wARP* (Langer *et al.*, 2008 ▸; Cohen *et al.*, 2008 ▸) and *Buccaneer* (Cowtan, 2006 ▸). The *AMPLE* approach has proved to be highly successful for coiled-coil structures, solving approximately 80% of a test set of 94 coiled-coil data sets, including structures of up to 253 amino acids and of resolutions as low as 2.9 Å (Thomas *et al.*, 2015 ▸), and has been used to solve a number of novel coiled-coil structures (Bruhn *et al.*, 2014 ▸; Hill *et al.*, 2017 ▸; Dunce *et al.*, 2018 ▸). An alternative approach is implemented by *ARCIMBOLDO*, in which small ideal helical fragments are placed by MR, with solutions expanded by density modification and helical tracing in *SHELXE*, and is highly successful for coiled-coil structure solution (Rodríguez *et al.*, 2009 ▸; Caballero *et al.*, 2018 ▸). Another approach has been provided by *CCsolve*, which generates oligomeric coiled-coil models for use as search templates in MR (Rämisch *et al.*, 2015*a* ▸).

Here, we address the 20% of the coiled-coil test set that *AMPLE* has hitherto failed to solve automatically. We report two new *Rosetta ab initio* model-building and treatment methods within *AMPLE* that greatly improve its ability to solve coiled-coil structures. Firstly, the imposition of elongated restraints in model building led to the production of more accurate models. Secondly, use of the *Rosetta Fold-and-Dock* protocol with unbiased coiled-coil restraints generated highly accurate oligomeric coiled-coil models that were processed into truncated ensembles for MR within *AMPLE*. A combination of general improvements in the *AMPLE* pipeline, the removal of tNCS correction in molecular replacement and these new modelling methods enabled the solution of 18 of the 22 previously unsolved coiled-coil test sets, including a case with 484 amino acids in the asymmetric unit at 2.8 Å resolution. These findings have been implemented in a new *AMPLE* coiled-coil mode, in which either elongated monomers or oligomeric coiled-coil models are generated and processed in a fully automated setting. We utilized the automated coiled-coil mode in *AMPLE* for oligomeric models to solve five new coiled-coil test cases of parallel dimers, trimers and tetramers at resolutions of between 3.0 and 3.3 Å, thus extending the solvability of coiled-coil structures by *AMPLE* to lower resolution cases.

## 2. Materials and methods   

### 2.1. Test data sets for coiled-coil structure solution   

The test set used for testing coiled-coil structure-solution methods consists of the 22 cases from the previous *AMPLE* coiled-coil test set that had failed to solve automatically, including three cases that were solved upon manual intervention (Thomas *et al.*, 2015 ▸). These are PDB entries 1g1j, 1gmj, 1m3w, 1s35, 1y66, 2efr, 2fxm, 2v71, 2wpq, 3azd, 3bas, 3cvf, 3h7z, 3hfe, 3mqc, 3q8t, 3s4r, 3t97, 3trt, 3v86, 3vir and 4dzk. An additional test set of lower resolution coiled-coil data sets was established to test the automated *AMPLE* coiled-coil multimeric modelling mode. These were identified by searching the Protein Data Bank for structures with resolutions between 3.0 and 3.3 Å and a helical content of >80%, with manual inspection to confirm the presence of parallel coiled-coil structure, and consist of PDB entries 3mqb, 3v4q, 4gkw, 4qkv, 4u5t, 6bri and 6gbr. Coiled-coil structure was analysed by *SOCKET* (http://coiledcoils.chm.bris.ac.uk/socket/server.html; Walshaw & Woolfson, 2001 ▸), with an ideal coiled-coil target defined by the presence of a single un­interrupted coiled-coil covering >50% of the protein sequence. Coiled-coil geometry was analysed by *TWISTER* (https://pharm.kuleuven.be/apps/biocryst/twister.php; Strelkov & Burkhard, 2002 ▸). The characteristics of the test data sets are summarized in Table 1 ▸ and Supplementary Data Set S1.

### 2.2. *AMPLE* pipeline and assessment of solutions   

*AMPLE* (Bibby *et al.*, 2012 ▸) runs were performed using version 1.4.6 of the software with *CCP*4 version 7.0.68. In the *AMPLE* pipeline, *ab initio* modelling was performed by *Rosetta* (Simons *et al.*, 1997 ▸, 1999 ▸, 2001 ▸) using *Robetta* fragment libraries generated with the exclusion of homologous structures (http://robetta.bakerlab.org). 1000 models were clustered using *SPICKER* (Zhang & Skolnick, 2004 ▸), and ensembles with polyalanine treatment were generated from ten clusters and up to 20 truncation levels at r.m.s.d. values of 1 and 3 Å. Structure solution was attempted for each ensemble via *MrBUMP* (Keegan & Winn, 2007 ▸; Keegan *et al.*, 2018 ▸) through molecular replacement using *Phaser* (McCoy *et al.*, 2007 ▸) and chain tracing by *SHELXE* (Sheldrick, 2015 ▸; Usón & Sheldrick, 2018 ▸). The correction of tNCS by *Phaser* was disabled by including the command -mr_keys PKEY TNCS USE OFF in the *AMPLE* run script. Solutions were ranked on the basis of their *SHELXE* correlation coefficients (CCs) and the top-ranked *SHELXE* solution was used for automated model building. This was performed using *Phenix AutoBuild* version 1.14-3260 (Liebschner *et al.*, 2019 ▸) with the option ‘build helices and strands only’ selected, using the *SHELXE* build as the initial model, tested with and without the use of NCS in density modification, with iterative runs until no further improvements were observed. Successful structure solution was determined by *R*_free_ values (<0.45), map quality (side-chain density *etc.*), completeness of the model (overall and side-chain placement) and the correlation coefficient between its 2*F*_o_ − *F*_c_ map and the deposited structure (>0.60), calculated by *phenix.get_cc_mtz_pdb*, with manual inspection of its origin-corrected superposition of solution, map and deposited structure. In cases where structure solution had failed, lower ranked *SHELXE* solutions of high CC were analysed to ensure that correct solutions had not been overlooked. Models and maps were inspected using *Coot* version 0.8.7 (Emsley *et al.*, 2010 ▸). Molecular-structure images were generated using *PyMOL* (version 2.0; Schrödinger).

### 2.3. Elongated monomeric model building within *AMPLE*   

*Rosetta* modelling within the *AMPLE* pipeline was modified to force the generation of elongated monomers by the removal of *R*_g_ weight scoring and the imposition of a long-distance restraint between the N- and C-terminus of the target sequence. The distance restraint was set at 1.5*n*, where *n* is the number of amino-acid residues corresponding to the well established α-helical rise of 1.5 Å per amino acid. This was achieved through the following *AMPLE* commands -rg_reweight 0 and -domain_termini_distance 1.5*n* (where *n* is the number of amino-acid residues).

### 2.4. Oligomeric coiled-coil modelling and use within *AMPLE*   

Oligomeric coiled-coil structures were modelled using the *Rosetta Fold-and-Dock* application (Das *et al.*, 2009 ▸; Rämisch *et al.*, 2015*b* ▸). Symmetry definition files for *C*2, *C*3 and *C*4 were generated by the *Rosetta* script make_symmdef_file_denovo, and were used for parallel dimers, trimers and tetramers, respectively. Modelling was performed using long-distance restraints between the N- and C-terminal residues and short-distance restraints between symmetry-related copies of the same amino acid, specified for C^α^ atoms. Distance restraints used the FLAT_HARMONIC function with a flat width of ±5 Å and 3 Å standard deviation, with central values of 1.5*n* (as described above) for long-distance restraints and 10 Å for short-distance restraints. The *Rosetta Fold-and-Dock* input flags are listed below.
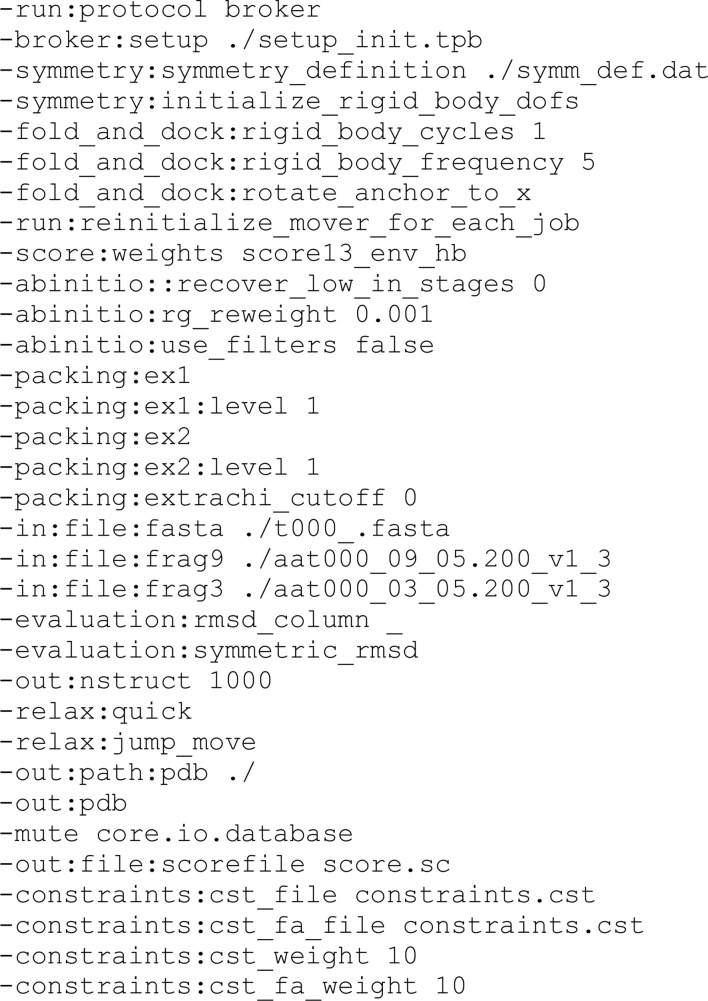


Distance restraints are defined within the *Rosetta* ‘constraints file’ (constraints.cst). An extract from an example file, showing long-distance (first line) and short-distance (lines 2–5) restraints, is included below.
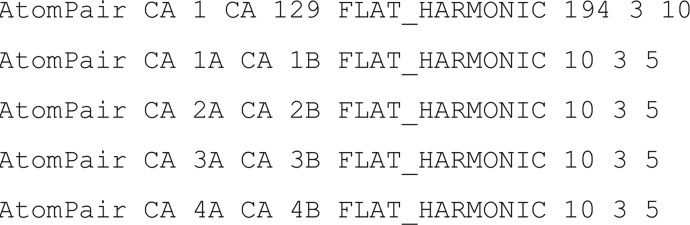


Oligomeric models were used in *AMPLE* by assigning consecutive numbering in chain *A* to all modelled symmetry-related chains using the *cctbx* library (Adams *et al.*, 2010 ▸), with a sequence file containing the appropriate number of copies of the input sequence. The resulting models were then handled appropriately in clustering, truncated ensemble generation and molecular replacement by the existing *AMPLE* pipeline. In cases where the asymmetric unit was smaller than the coiled-coil oligomer, the models were first truncated to the appropriate number of chains before consecutive renumbering (for example, only chains *A* and *B* were processed for a tetramer with two chains in the asymmetric unit, such that a ‘half-tetramer’ was used in molecular replacement).

### 2.5. *AMPLE* coiled-coil mode   

The above commands for generating and using elongated monomeric and oligomeric coiled-coil models within *AMPLE* have been automated in a new ‘coiled-coil’ mode. This is included in *AMPLE* version 1.5.0, which is available at https://github.com/linucks/ample.git and will be part of *CCP*4 version 7.1 and subsequent versions. Coiled-coil mode is activated by the flag -coiled_coil, which automatically imposes elongated monomeric helical modelling (as described above) and disables tNCS correction in *Phaser*. Oligomeric coiled-coil modelling is activated by the flag -multimer_modelling [dimer|trimer|tetramer], with asymmetric unit contents defined by the flag -nmasu [number of chains in asymmetric unit], which automatically uses the methods described above to generate and process oligomeric coiled-coil models within the *AMPLE* pipeline.

## 3. Results and discussion   

### 3.1. Characteristics of the coiled-coil test set   

Of the original *AMPLE* coiled-coil test set of 94 targets, almost 80% were solved automatically (Thomas *et al.*, 2015 ▸), leaving 22 unsolved data sets (including three that were solved manually) that are the focus of this study. These include parallel/antiparallel dimers, trimers and tetramers, and a variety of triclinic, monoclinic, orthorhombic, trigonal and hexagonal space groups, at resolutions between 0.98 and 2.91 Å (Table 1 ▸ and Supplementary Data Set S1). We analysed the presence of ideal ‘knobs-into-holes’ structures using *SOCKET* (Walshaw & Woolfson, 2001 ▸). On the criterion that an ideal coiled-coil target contains a single uninterrupted coiled-coil covering >50% of its sequence, nine out of the 22 targets are ideal coiled-coil targets (Table 1 ▸ and Supplementary Data Set S1). The remaining 13 targets contain interrupted and/or overlapping coiled-coils, structural deviation from ‘knobs-into-holes’ packing and/or more complicated geometries/topologies. Coiled-coil geometry analysis by *TWISTER* (Strelkov & Burkhard, 2002 ▸) showed that the test set includes 17 left-handed and two right-handed coiled-coils, two that transition between right-handed and left-handed along their length (PDB entries 3h7z and 3trt) and one α-helical monomer (PDB entry 1s35) (Table 1 ▸). The range of inter-helical crossing angles is between 14° and 37° for left-handed coiled-coils and 9° and 25° for right-handed coiled-coils (Supplementary Data Set S1). Thus, the test set represents a range of structurally diverse and non-ideal coiled-coils.

### 3.2. General improvements to the *AMPLE* pipeline   

**Figure 1:**
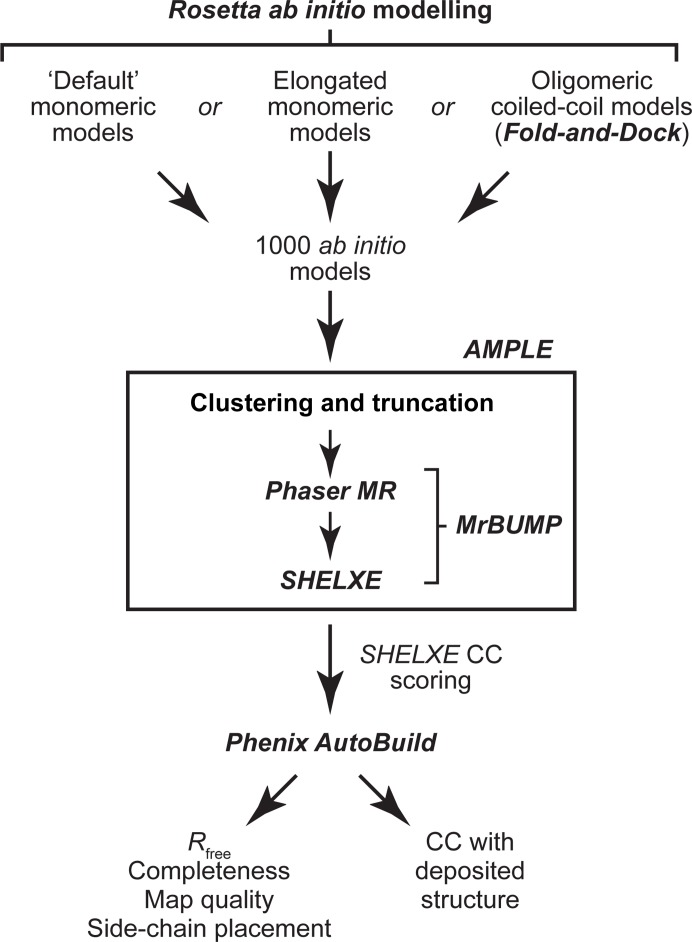
Summary of the approaches utilized for coiled-coil structure solution by *AMPLE* in this study. The *AMPLE* pipeline generated a series of clustered and truncated ensembles for each of the three *ab initio* modelling methods (each clustered from 1000 models), which were fed into the *MrBUMP* pipeline for *Phaser* molecular replacement followed by *SHELXE* chain tracing. Automated structure building was performed using *Phenix AutoBuild* for the highest *SHELXE* correlation coefficient solution of each modelling method. Successful solution was determined by the *R*_free_ (<0.45), build completeness, map quality and correlation coefficient (>0.60) with the deposited structure.

We first established the new *AMPLE* baseline, accounting for general improvements in *AMPLE* and its utilized programs, by performing automated runs with default settings for unsolved test cases. Our experiences of using *AMPLE* to solve novel coiled-coil structures in previous (Dunce *et al.*, 2018 ▸) and unpublished work have consistently shown success from density modification and autotracing of *Phaser* solutions in *SHELXE* (Sheldrick, 2015 ▸; Usón & Sheldrick, 2018 ▸), followed by automated model building of only helices and strands by *Phenix AutoBuild* (Liebschner *et al.*, 2019 ▸). We thus established a pipeline for *AMPLE* runs in which the top-ranked *SHELXE* build, according to its correlation coefficient (CC), was subjected to iterative building rounds by *Phenix AutoBuild* (Fig. 1 ▸). Its success or failure was determined both *a priori* (independent of the deposited structure) and through direct comparison with the deposited structure. In general, successful solutions are associated with *Phaser* LLG and TFZ scores of >120 and >8, respectively (McCoy *et al.*, 2007 ▸), and in *AMPLE* by a *SHELXE* CC of >25 and a model *R*_free_ of <0.45 (Thomas *et al.*, 2015 ▸). However, these values are less reliable at lower resolutions and are further confounded by the unusual characteristics of coiled-coils. Indeed, we encountered incorrect solutions in this study with *Phaser* LLG >500 and TFZ >10 and with a *SHELXE* CC of >50 and an *R*_free_ of <0.42 (Table 1 ▸ and Supplementary Data Set S1). Thus, we suggest that no single metric should be used to gauge the success of coiled-coil structure solution *a priori*, but it should instead be judged by a combination of high *SHELXE* CC (>30 and a difference of 5–10 between the top and background solutions), low *R*_free_ (<0.45), high model build completeness, map quality and placement of side chains during model building. In parallel, we assessed the success of solutions by a correlation coefficient of >0.60 between the 2*F*_o_ − *F*_c_ map and the deposited structure, as calculated by *phenix.get_cc_mtz_pdb* (Adams *et al.*, 2010 ▸), alongside manual inspection of the superimposed map, model and deposited structure. Of the 22 previously unsolved test coiled-coil cases, eight (PDB entries 1gmj, 1m3w, 1y66, 3bas, 3q8t, 3s4r, 3t97 and 4dzk) were solved by *AMPLE*, indicating that improvements in the pipeline, and in *Phaser* and *SHELXE* (Usón & Sheldrick, 2018 ▸), since the original analysis have enhanced coiled-coil structure solution by *AMPLE*.

### 3.3. Aberrant tNCS correction can preclude coiled-coil solution   

**Figure 2:**
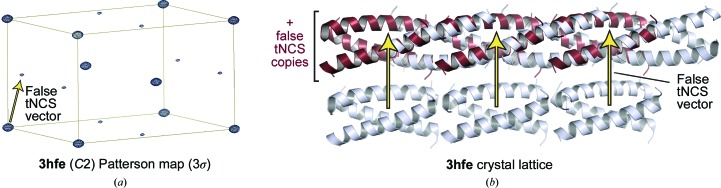
Detection of a false tNCS (translational noncrystallographic symmetry) vector for PDB entry 3hfe. (*a*) Patterson map (contoured at 3σ) showing a non-origin peak at 21% of the origin peak height that led to identification of the false tNCS vector. (*b*) Crystal lattice (light blue) superimposed with molecules translated by the false tNCS vector (red), highlighting that it arose through apparent similarity between symmetry-related copies (with opposite orientation) in adjacent chains owing to the recursive nature of the coiled-coil structure.

In previous work, we have observed that the internal symmetry of coiled-coils frequently gives rise to non-origin Patterson peaks owing to correlations between symmetry-related copies that are often in reverse orientation (Dunce *et al.*, 2018 ▸). These are interpreted by *Phaser* as originating from translational noncrystallographic symmetry (tNCS) between asymmetric unit components, leading to correction for data modulation and MR using components linked by the tNCS vector. Whilst beneficial in most cases, this is detrimental if detected erroneously for coiled-coils. We observed for a number of unpublished cases that disabling tNCS correction was essential for successful structure solution, which has also been reported for *ARCIMBOLDO* (Caballero *et al.*, 2018 ▸). We noticed that tNCS correction had been performed for three structures (PDB entries 2wpq, 3hfe and 4dzk), and so we repeated these runs with tNCS correction disabled through the *AMPLE* command -mr_keys TNCS USE OFF. This resulted in the solution of PDB entries 2wpq and 3hfe, which were previously unsolved, and improved the solution of PDB entry 4dzk (Table 1 ▸ and Supplementary Data Set S1). Analysis of PDB entry 3hfe demonstrated that tNCS was detected owing to the similarity of the recursive coiled-coil structure, with imposition of the false tNCS vector leading to the superposition of molecules onto symmetry-related copies in reverse orientation (Figs. 2 ▸ *a* and 2 ▸ *b*). On the basis of this and our previous observations, we suggest that coiled-coil structure solution should be run in the first instance with tNCS correction disabled, as implemented in the coiled-coil mode of *ARCIMBOLDO* (Caballero *et al.*, 2018 ▸).

### 3.4. Structure solution from elongated α-helical models   

**Figure 3:**
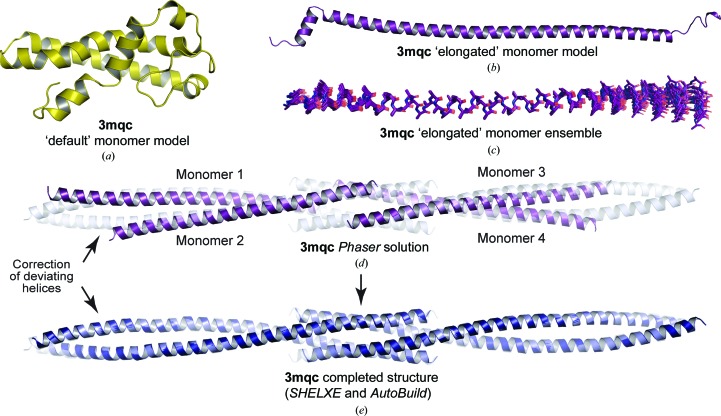
Structure solution of PDB entry 3mqc using the *AMPLE* elongated monomer modelling method. (*a*, *b*) Top-scoring *Rosetta* *ab initio* models of PDB entry 3mqc using (*a*) the default monomer modelling method and (*b*) the elongated monomer modelling method. (*c*) The clustered and truncated ensemble of elongated monomer models that was successful in structure solution. (*d*, *e*) Structure solution of PDB entry 3mqc showing (*d*) the *Phaser* solution (purple) in which four elongated monomeric models were placed and (*e*) the completed structure (blue) following *SHELXE* chain tracing and automated building by *Phenix AutoBuild*; the deposited structure is shown in grey.

What is limiting the ability of *AMPLE* to solve the remaining 12 structures of the coiled-coil test set? *Ab initio* models generated by *Rosetta* for coiled-coils are monomeric and typically globular (see, for example, Fig. 3 ▸ *a*), so differ substantially from native oligomeric coiled-coils, with successful solution relying on conformational similarity between helical fragments within globular models and part of the coiled-coil structure. We reasoned that by forcing *Rosetta* to build nonglobular elongated models we would increase the chance of generating large fragments that match the native structure, and thereby increase the chance of structure solution. We thus implemented a modified *Rosetta* modelling procedure in which the *R*_g_ weighting was eliminated to minimize the bias towards globularity (-rg_reweight 0), and a long-distance constraint was imposed between the N- and C-termini to enforce an elongated build (-domain_termini_distance 1.5*n*). The distance constraint was selected as 1.5*n*, where *n* is the number of amino-acid residues in the sequence, given the well established rise of 1.5 Å per amino acid for an α-helical structure (Truebestein & Leonard, 2016 ▸). This led to the consistent generation of elongated helical models for all coiled-coil sequences tested (see, for example, Fig. 3 ▸ *b*), taking the same length of time to generate models as in default modelling (for example, PDB entry 3v86 models were generated at an average of 33 and 32 s per model for the default and elongated procedures, respectively). Importantly, these commands did not enforce helicity, so this was generated by *Rosetta* on the basis of the fragment library, with the helical conformation determined by the physicochemical properties of the sequence. We repeated *AMPLE* runs on all test cases using this new ‘elongated’ models mode. This led to the solution of five new structures (PDB entries 2v71, 3cvf, 3h7f, 3mqc and 3trt) in addition to all of the structures that had previously been solved by ‘default’ models. As an example, PDB entry 3mqc failed to solve using default models (Fig. 3 ▸ *a*), but was successfully solved using elongated models (Fig. 3 ▸ *b*), through an ensemble that included substantial common helical structure with deviation at either end (Fig. 3 ▸ *b*). *Phaser* placed four search models in the asymmetric unit (Fig. 3 ▸ *d*), and subsequent automated building by *SHELXE* and *Phenix AutoBuild* led to correction of helical deviations with a completed model that matches the deposited structure (Fig. 3 ▸ *e*). In cases that solved using both methods, we observed a higher percentage of successful solutions for elongated models than for default models, meaning that the first successful solution was typically obtained more quickly, requiring less CPU time. On this basis, and given that elongated modelling takes no more CPU time per model than default modelling, we propose that elongated modelling should be used as standard for coiled-coil structure solution by *AMPLE*.

### 3.5. Structure solution from oligomeric coiled-coil models   

**Figure 4:**
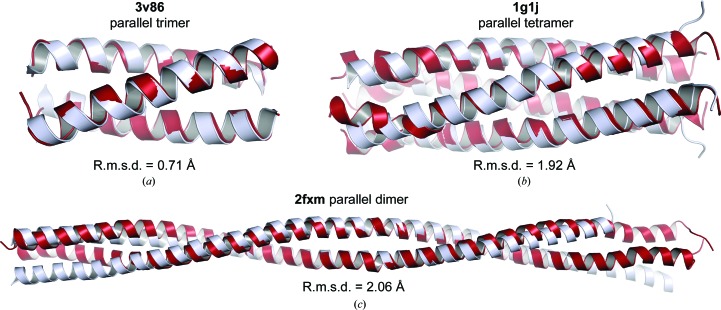
Generation of accurate oligomeric coiled-coil models using *Rosetta Fold-and-Dock*. (*a*, *b*, *c*) Top-scoring *Rosetta* oligomeric models (red) with the deposited structures superposed (grey) for (*a*) a parallel trimer from PDB entry 3v86, (*b*) a parallel tetramer from PDB entry 1g1j and (*c*) a parallel dimer from PDB entry 2fxm; the all-atom r.m.s. deviation between the model and the deposited structure is shown.

Our elongated modelling approach still left seven targets in the test set unsolved. We reasoned that further improvements would require higher accuracy of the helical conformation and/or the placement of larger fragments, both of which could be achieved by modelling full oligomeric coiled-coils. Whilst highly accurate oligomeric models of ideal coiled-coils can be generated rapidly by parameterization or threading-based methods (Wood & Woolfson, 2018 ▸; Guzenko & Strelkov, 2017 ▸), it remains extremely challenging to accurately predict the non-ideal geometries and topologies that are widely observed within the coiled-coil family (Lupas *et al.*, 2017 ▸; Moutevelis & Woolfson, 2009 ▸). We thus exploited the wide conformational sampling within *Rosetta* such that models would be built based entirely on the physiochemical properties of their amino-acid sequence, without bias towards ideal coiled-coil structure. We established a method for modelling oligomeric coiled-coils using the *Rosetta Fold-and-Dock* protocol, in which one chain is modelled around a symmetry axis (Das *et al.*, 2009 ▸; Rämisch *et al.*, 2015*b* ▸). Using challenging targets from SYCP1 (Dunce *et al.*, 2018 ▸), we found that the inclusion of distance restraints was essential to achieve a high percentage of successful builds such that 1000 models would be sufficient for generating useful ensembles. We developed an unbiased method for coiled-coil modelling through the use of long-distance and short-distance restraints, with *C*2, *C*3 and *C*4 symmetry used to specify parallel dimers, trimers and tetramers, respectively. The long-distance restraints define the N- and C-terminal separation as 1.5*n* (as described in Section 3.3). The short-distance restraints are between symmetry-related copies of C^α^ atoms of the same residue, imposing no penalty between 5 and 15 Å (encompassing both internal heptad/hydrophobic interactions and external solvent-exposed distances), with penalties imposed outwith this range with a standard deviation of 3 Å. These loose restraints proved sufficient to direct coiled-coil modelling, whilst providing no user-based bias regarding which amino acids form the heptad (or other periodicity) or regarding coiled-coil geometry. We tested this method using the unsolved test cases, and the top-scoring models (based on the *Rosetta* energy function) closely matched the deposited structures of PDB entries 1g1j, 2fxm and 3v86, with r.m.s.d. values between 0.71 and 2.06 Å (Figs. 4 ▸ *a*, 4 ▸ *b* and 4 ▸ *c*). Further, oligomeric modelling took only approximately four times longer than default or elongated modelling (for example, oligomers for PDB entry 3v86 were generated in 144 s per model, compared with 33 s for default monomers) and so is achievable within a reasonable timeframe on a standard computer (for example, 1000 oligomeric models of PDB entry 3v86 could be generated on a 12-core processor in just over 3 h).

**Figure 5:**
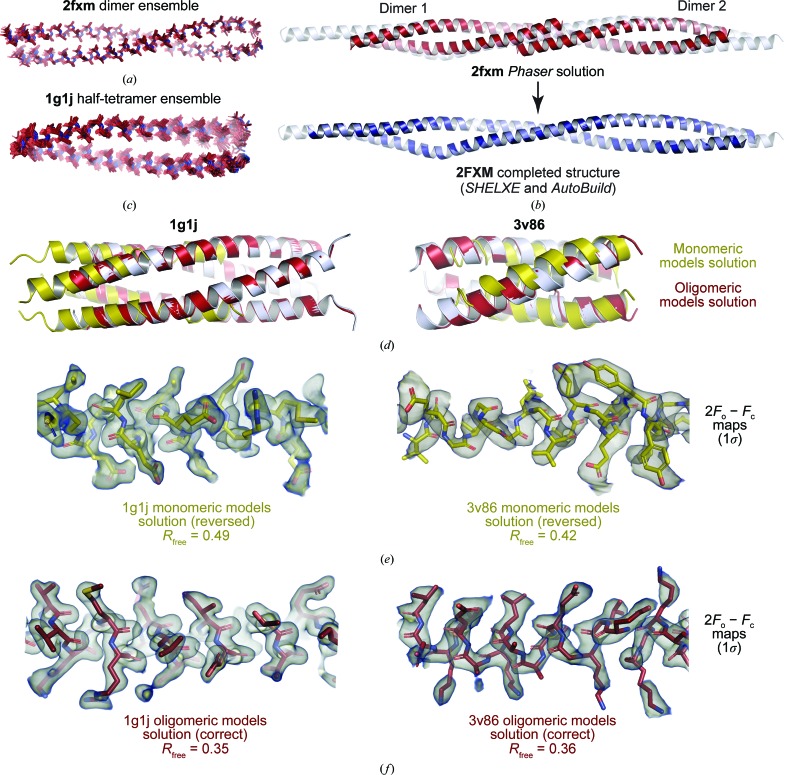
Structure solution using the *AMPLE* oligomeric coiled-coil modelling method. (*a*) The clustered and truncated ensemble of PDB entry 2fxm dimer models that was successful in structure solution. (*b*) Structure solution of PDB entry 2fxm showing the *Phaser* solution (red) in which two dimeric models were placed and the completed structure (blue) following *SHELXE* chain tracing and automated building by *Phenix AutoBuild*; the deposited structure is shown in grey. (*c*) Clustered and truncated ensemble of PDB entry 1g1j half-tetramers (modelled as tetramers and reduced to two chains for molecular replacement) that was successful in structure solution. (*d*, *e*, *f*) Structure solution of PDB entries 1g1j and 3v86 using oligomeric coiled-coil models, with an incorrect solution using monomeric models shown for comparison. (*d*) Superposition of the oligomeric model solution (red), monomeric model solution (yellow) and deposited structure (grey) for PDB entries 1g1j (left) and 3v86 (right). (*e*, *f*) 2*F*_o_ − *F*_c_ maps (contoured at 1σ) for (*e*) monomeric model solutions in which chains were incorrectly placed backwards and (*f*) correct oligomeric model solutions, showing their *R*_free_ values, for PDB entries 1g1j (left) and 3v86 (right).

We implemented the use of oligomeric models in *AMPLE* by reducing models down to the number of chains within the asymmetric unit (when less than the oligomer state) and reorganizing oligomers as consecutive sequences within a single chain to allow their use in clustering and ensembling. This led to the successful solution of three (PDB entries 1g1j, 2fxm and 3v86) of the remaining seven structures. These cases involved ensembling and placement of the full dimer for PDB entry 2fxm (Fig. 5 ▸ *a*), a half-tetramer consisting of two chains from the modelled tetramer for PDB entry 1g1j (Fig. 5 ▸ *b*) and a single chain from the modelled trimer for PDB entry 3v86 (Table 1 ▸ and Supplementary Data Set S1). In the case of PDB entry 2fxm, the dimer was placed twice in the asymmetric unit to create the larger overall dimeric structure, with deviation of the placed molecules corrected by subsequent model building in *SHELXE* and *Phenix AutoBuild* (Fig. 5 ▸ *c*).

The structures with PDB codes 1g1j and 3v86 comprise cases in which default and elongated models produced plausible solutions that were incorrect, but were correctly solved using oligomeric models. In both cases, default and elongated models were placed in reverse orientations (Fig. 5 ▸ *d*) but generated reasonable 2*F*_o_ − *F*_c_ electron-density maps (Fig. 5 ▸ *e*). Whilst the 2*F*_o_ − *F*_c_ maps of the correctly placed oligomeric models were clearly superior (Fig. 5 ▸ *f*), this was only apparent *a posteriori*. In the case of PDB entry 1g1j, the high *R*_free_ (>0.49) and the inability to place any side chains during model building should have raised alarms despite any obvious signs from 2*F*_o_ − *F*_c_ or *F*_o_ − *F*_c_ maps (Table 1 ▸ and Supplementary Data Set S1). However, the relatively low *R*_free_ (<0.42), following a *SHELXE* CC of 51.7, for the incorrect and reversed orientation of PDB entry 3v86 highlights the need to assess all statistics in judging whether or not a solution is correct; in this case, the high *R*–*R*_free_ gap (>0.12) and the failure to place any side chains were obvious causes for concern (Figs. 5 ▸ *d*, 5 ▸ *e* and 5 ▸ *g*, Table 1 ▸ and Supplementary Data Set S1).

Of the remaining unsolved cases, PDB entry 1s35 has a spectrin-repeat structure that would be unlikely to benefit from our new coiled-coil modelling methods and PDB entry 3vir forms a higher-order structure within the crystal lattice lacking a clear oligomer that could be modelled. Further, whilst PDB entry 3azd is a simple parallel dimer, the experimental data lack low-resolution reflections and the reported measurement errors are unusually low, which is likely to have prevented solution, as discussed previously (Caballero *et al.*, 2018 ▸). The remaining structure, PDB entry 2efr, consists of two long parallel dimers in the asymmetric unit of 620 amino acids, which we could not solve using *AMPLE* despite generating oligomeric models containing accurate regions relative to the deposited structure.

The three successful oligomeric modelling solutions suggest that enhanced structure solution was achieved at two levels. Firstly, the higher accuracy of oligomeric modelling increased the likelihood of the correct placement of individual chains. Secondly, the use of oligomeric search models increased the chance of correct placement during rotation and translation, owing to the larger signal of the oligomer and fixed orientation between helices, reducing the risk of reverse placement and/or register errors including placement across symmetry-related copies. On the basis of our findings, we propose the use of oligomeric models in *AMPLE* when elongated models fail and in cases of lower resolution or crystallographic pathologies when the higher accuracy, increased size and fixed orientation of helices within oligomeric models can aid discrimination between correct and incorrect solutions.

### 3.6. A new automated ‘coiled-coil’ mode for *AMPLE*   

We next implemented our findings in a new ‘coiled-coil’ mode for *AMPLE*. This is activated by a single flag (-coiled_coil) that automatically executes elongated modelling (as described in Section 3.3) and disables tNCS correction. Alternatively, oligomeric modelling may be activated within coiled-coil mode by specifying the parallel oligomer type (-multimer_modelling [dimer|trimer|tetramer]) and asymmetric unit contents (-nmasu [number of chains per asymmetric unit]). This implements the *Rosetta Fold-and-Dock* protocol (described in Section 3.4), with *C*2, *C*3 or *C*4 symmetry and an automatically generated constraints file, and reduces oligomers (if necessary) based on the defined asymmetric unit contents for the clustering and generation of ensembles for molecular replacement within the *AMPLE* pipeline.

**Figure 6:**
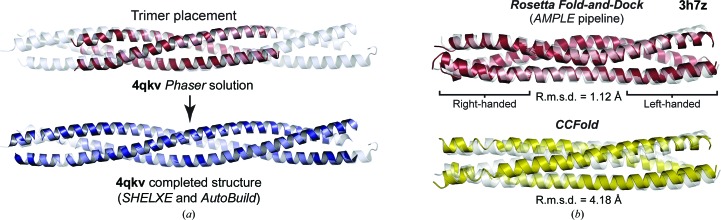
Automated oligomeric modelling and structure solution by the coiled-coil mode of *AMPLE*. (*a*) Structure solution of PDB entry 4qkv. The *Phaser* solution (purple) is shown in which a single trimer was placed alongside the completed structure (blue) following *SHELXE* chain tracing and automated building by *Phenix AutoBuild*; the deposited structure is shown in pale blue. (*b*) Top-scoring oligomeric model of PDB entry 3h7z generated by *Rosetta Fold-and-Dock* through the automated *AMPLE* pipeline (red, top) and the model of PDB entry 3h7z generated by *CCFold* (yellow, bottom). The deposited structure is shown in grey and model r.m.s.d. values are indicated.

We tested the new coiled-coil mode of *AMPLE* with five test cases of parallel dimers, trimers and tetramers, consisting of one ideal and four non-ideal coiled-coil targets, with left-handed super-helical crossing angles between 24° and 30°, at resolutions between 3.0 and 3.3 Å (Table 1 ▸ and Supplementary Data Set S1). The fully automated oligomeric coiled-coil mode produced clear-cut solutions for three cases, with the placement of a half-dimer (one chain of a modelled dimer) for PDB entry 3v4q, a full dimer for PDB entry 4gkw and a full trimer for PDB entry 4qkv (Fig. 6 ▸ *a*), along with a borderline solution for dimeric PDB entry 4u5t. We had noticed that *Rosetta* modelling scores (based on its energy function) are closely correlated with model accuracy, so we wondered whether we could improve structure solution by restricting *AMPLE* to using only the 25 top-scoring oligomeric models. This led to clear-cut solutions for PDB entry 4u5t and the previously unsolved PDB entry 6gbr, the latter case involving the placement of a full tetramer in the asymmetric unit (Table 1 ▸ and Supplementary Data Set S1). Thus, the new oligomeric coiled-coil mode of *AMPLE* successfully solved five challenging cases at resolutions below 3.0 Å.

The automated coiled-coil mode is available within the latest build of *AMPLE* and is distributed within the *CCP*4 software suite. The coiled-coil mode may be activated for elongated or oligomeric modelling using the flags described.

## 4. Conclusions   

Here, we describe an enhanced functionality for *AMPLE* in coiled-coil structure solution through improvements in the *ab initio* modelling of coiled-coils as either elongated monomeric α-helices or as full oligomers, which we have implemented in a new automated ‘coiled-coil’ mode.

**Figure 7:**
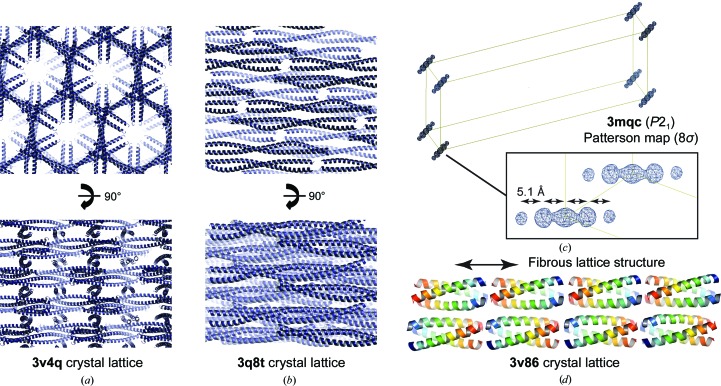
Summary of crystal lattice characteristics observed for coiled-coil structures. (*a*, *b*) Crystal lattices of PDB entries 3v4q and 3q8t demonstrating clearly demarcated individual coiled-coil molecules arranged (*a*) in an angulated lattice with high solvent content and (*b*) in a densely packed parallel array with low solvent content. (*c*) The Patterson map (contoured at 8σ) of PDB entry 3mqc showing linear arrangements of peaks separated by 5.1 Å that arise from the 5.1 Å coiled-coil repeat owing to its packing in a parallel array, such as shown in (*b*), and indicate the direction of the principal coiled-coil axis. (*d*) The crystal lattice of PDB entry 3v86 in which trimeric coiled-coils are arranged in fibre-like structures that overall resemble individual continuous trimeric coiled-coils of unlimited length.

Our analyses of coiled-coil structures in this and previous studies highlight features and pathologies that commonly occur in coiled-coil protein crystals, and are in agreement with those described by others (Guzenko *et al.*, 2017 ▸; Blocquel *et al.*, 2014 ▸; Dauter, 2015 ▸; Caballero *et al.*, 2018 ▸). In some cases, coiled-coils crystallize with a high angulation and large solvent channels, providing a favourable configuration for placement by MR and/or the location of heavy atoms in experimental phasing (Fig. 7 ▸ *a*). However, it is seemingly more common for coiled-coils to crystallize in densely packed parallel arrays with low solvent content (Fig. 7 ▸ *b*). In this latter configuration, preferential orientation along a common axis means that linear arrays of Patterson peaks separated by 5.1 Å, which arise from the helical pitch along the coiled-coil axis, are orientated in the same direction and so become extremely prominent and reveal the coiled-coil axis orientation (Fig. 7 ▸ *c*). These Patterson features, together with apparent tNCS between symmetry-related copies and the low contrast between protein and solvent, pose significant challenges for structure solution by both MR and experimental phasing. Further, whilst such densely packed lattices often consist of clearly demarcated oligomers, in some cases coiled-coils line up to form fibres that resemble single super-helical structures of indefinite length (Fig. 7 ▸ *d*). This poses additional problems in molecular replacement as the difference in agreement between correct/incorrect solutions and the experimental data is often minimal. The ability of structure solution depends on a balance of model quality, crystal quality and resolution. Thus, whilst poor models may be sufficient for solution in favourable high-resolution cases, more accurate models, such as those obtained through the elongated or oligomeric modelling procedures in *AMPLE*, may be required at lower resolution and/or for weak data or crystal pathologies/characteristics as outlined above.

The coiled-coil test set used in this study encompasses a diverse range of parallel/antiparallel dimers, trimers and tetramers, consisting of left-handed and right-handed coiled-coil structures with a variety of inter-helical crossing angles and non-ideal geometries and topologies. This includes four non-ideal coiled-coil targets that were solved using oligomeric models, suggesting that the *Rosetta Fold-and-Dock* method employed by *AMPLE* is suitable for the diverse non-ideal structures exhibited by coiled-coils. To confirm this, we tested whether *AMPLE* could generate accurate models of the most unusual target within the test set, PDB entry 3h7z, which is a parallel trimer that undergoes a full transition along its length from a right-handed coiled-coil (crossing angle of 25°) to a left-handed coiled-coil (crossing angle of 35°). The top-scoring model (using the *Rosetta* modelling score) out of 100 models generated by the fully automated oligomeric coiled-coil mode in *AMPLE* clearly replicated the right-handed to left-handed coiled-coil transition and closely matched the deposited PDB entry 3h7z structure (r.m.s.d. of 1.12 Å; Fig. 6 ▸ *b*). In contrast, a structural model generated by a threading-based method in *CCFold* (Guzenko & Strelkov, 2017 ▸) produced a continuous left-handed coiled-coil (r.m.s.d. of 4.18 Å; Fig. 6 ▸ *b*). Thus, the wide conformational sampling of *Rosetta Fold-and-Dock* appears to be suited to the diverse geometries and topologies of the coiled-coil family and exhibits clear advantages over faster methods based on prior knowledge of coiled-coil structure.

Whilst *AMPLE*’s oligomeric ‘coiled-coil’ mode requires prior knowledge of oligomer state and helical orientation, we have previously established that such information may be obtained by light and X-ray scattering experiments. The absolute oligomeric state of a coiled-coil may be accurately determined by SEC-MALS, and its orientation can be established by SEC-SAXS analysis of proteins harbouring N- or C-terminal MBP fusions (Dunce *et al.*, 2018 ▸; Dunne & Davies, 2019*a* ▸,*b* ▸). Thus, oligomeric modelling may be guided by biophysical analysis. Further, a clear advantage of the approach in *AMPLE* is the relatively low CPU time required for each run, with full coiled-coil model building and the *AMPLE* pipeline typically requiring 1–2 days on a modest desktop computer. Thus, in situations in which the oligomeric state is uncertain, it is feasible to perform parallel runs in multiple oligomer states. Similarly, this permits the parallel analysis of multiple alternative space groups, as a more exhaustive method than testing all space groups in *Phaser*, using common elongated or oligomeric models. This is frequently necessary as internal symmetry and apparent tNCS within coiled-coil data sets can affect reflection intensities in a manner that obfuscates space-group determination from systematic absences. The current lack of support for antiparallel coiled-coils is owing to the complication of register ambiguity, which requires the use of more bespoke restraints to explore a number of alternative antiparallel registers. Nevertheless, antiparallel and higher order models may be generated separately by *Rosetta Fold-and-Dock*, using the restraints strategy described here, and then run through the *AMPLE* pipeline in coiled-coil mode using the ‘existing models’ option.

The generation of plausible but incorrect solutions for two cases in this study (PDB entries 1g1j and 3v86) when using default or elongated models (Figs. 5 ▸ *d*, 5 ▸ *e* and 5 ▸ *f*) raises the question of how we should accurately assess the success or failure of a coiled-coil solution at lower resolution. Whilst we found that no single metric is sufficient in isolation, a combination of a high *SHELXE* CC (>30 and 5–10 above background), a low *R*_free_ (<0.45), a low *R*–*R*_free_ gap (<0.05), model build completeness, map quality and placement of side chains during automated model building can be used to accurately assess the correctness of a solution. We find that the unique features and pathologies of each crystal form provide baseline statistics for incorrect solutions that can be markedly different for different crystals even at the same resolution. Thus, the most important assessment is to compare top and background solutions for a particular case, as true solutions tend to show clear demarcation in *SHELXE* CC and other statistics. This is particularly apparent if *AMPLE* is run in multiple alternative space groups, as a correct solution in the true space group should be clearly distinguished from solutions in incorrect space groups. We further recommend analysing a number of top solutions, which may be combined through origin correction using *phenix.get_cc_mtz_pdb*, as correct solutions will agree whereas multiple plausible incorrect solutions will typically differ.

The approach of model building and clustering/ensembling by *AMPLE* uses relatively large search models to maximize the signal in MR rotation and translation functions. In contrast, *ARCIMBOLDO* uses a different but highly successful approach of utilizing the consecutive placement of small but accurate helical fragments to gain initial phasing information (Caballero *et al.*, 2018 ▸). In comparing the ‘coiled-coil’ modes of *AMPLE* and *ARCIMBOLDO*, both solved all but four of the original *AMPLE* coiled-coil test set (Caballero *et al.*, 2018 ▸; Thomas *et al.*, 2015 ▸). Whilst one failed solution overlaps (PDB entry 3azd), the other three are different as *AMPLE* solved PDB entries 2fxm, 3mqc and 3s4r, whereas *ARCIMBOLDO* solved PDB entries 1s35, 2efr and 3vir (Caballero *et al.*, 2018 ▸). This agrees with our wider observations of differential successes for challenging cases in *AMPLE* and *ARCIMBOLDO*, highlighting the importance of differing approaches to the same problem. Thus, the enhanced capability of *AMPLE* in coiled-coil structure solution described here adds to the toolkit available to macromolecular structural researchers, maximizing the likelihood of achieving rapid structure solution from coiled-coil data sets in the absence of clear homologues or experimental phasing information, through an automated mode that requires minimal user input.

## Supplementary Material

Supplementary Data Set S1: a summary of the molecular-replacement and model-building statistics obtained for the coiled-coil test set using AMPLE. DOI: 10.1107/S2059798320000443/rr5190sup1.xlsx

## Figures and Tables

**Table 1:** Characteristics of the test data sets used in this study with a summary of the top solution statistics for all *AMPLE* default, elongated and oligomeric coiled-coil modelling methods Statistics are shown for *Phenix AutoBuild* models resulting from the highest *SHELXE* correlation coefficient solutions for each *AMPLE* method; bold indicates successful solution. The previously failed data sets of the *AMPLE* coiled-coil test set are shown in roman (Thomas *et al.*, 2015 ▸); new test data sets are shown in italics. An ideal coiled-coil is defined here through the detection by *SOCKET* (Walshaw & Woolfson, 2001 ▸) of a single uninterrupted coiled-coil covering more than 50% of the protein sequence (Supplementary Data Set S1). L and R, left-handed and right-handed coiled-coil. ASU, asymmetric unit.

PDB code	Resolution (Å)	Oligomer (parallel/antiparallel)	Ideal coiled-coil?	Chains per ASU	Residues per ASU	Autobuilt structures from top *SHELXE* solution (*R*_free_/CC between map and PDB entry)				
						Default models	Default models −tNCS	Elongated models	Elongated models −tNCS	Oligomeric models
1gmj	2.23	Dimer (a) L	No	4	336	**0.36/0.77**		**0.35/0.78**		
1m3w	2.80	Tetramer (a) R	No	4	128	**0.42/0.63**		**0.43/0.71**		
1y66	1.65	Tetramer (a) R	No	4	208	**0.35/0.83**		**0.35/0.82**		
3bas	2.31	Dimer (p) L	Yes	2	178	**0.41/0.74**		**0.41/0.74**		
3q8t	1.90	Dimer (a) L	Yes	2	192	**0.26/0.88**		**0.25/0.88**		
3s4r	2.45	Tetramer (a) L	No	2	186	**0.35/0.82**		**0.35/0.82**		
3t97	2.76	Trimer (p) L	Yes	3	192	**0.35/0.79**		**0.38/0.77**		
4dzk †	1.79	Trimer (p) L	Yes	1	32	**0.43/0.68**	**0.43/0.71**	**0.41/0.75**	**0.39/0.73**	
2wpq †	1.85	Trimer (p) L	Yes	3	297	0.52/0.04	**0.44/0.68**	0.52/0.03	**0.34/0.85**	
3hfe †	1.69	Trimer (p) L	No	3	93	0.51/0.04	**0.29/0.81**	0.55/0.10	**0.28/0.81**	
2v71	2.24	Tetramer (a) L	No	2	378	0.54/0.07		**0.34/0.80**		
3cvf	2.90	Tetramer (a) L	No	4	316	0.45/0.65		**0.36/0.82**		
3h7z	2.51	Trimer (p) R+L	No	1	61	0.49/0.27		**0.38/0.68 **		
3mqc	2.80	Tetramer (a) L	Yes	4	484	0.49/0.09		**0.37/0.78**		
3trt	2.30	Dimer (p) R+L	No	2	154	0.53/0.02		**0.36/0.84**		
1g1j	1.86	Tetramer (p) L	Yes	2	86	0.49/0.09		0.51/0.03		**0.35/0.83**
2fxm	2.70	Dimer (p) L	Yes	2	258	0.51/0.02		0.54/0.03		**0.36/0.83 **
3v86	2.91	Trimer (p) L	Yes	1	27	0.42/0.02		0.50/0.13		**0.36/0.67**
2efr	1.80	Dimer (p) L	No	4	620	0.52/0.06		0.54/0.06		0.52/0.11
3azd	0.98	Dimer (p) L	No	2	74	0.51/0.03		0.51/0.03		0.53/0.03
1s35	2.40	Monomer	No	1	214	0.54/0.03		0.54/0.02		N/A
3vir	2.70	Dimer (a) L	No	4	340	0.48/0.08		0.47/0.07		N/A
*3v4q*	3.06	Dimer (p) L	No	1	74					**0.37/0.81**
*4gkw*	3.30	Dimer (p) L	No	2	334					**0.35/0.75**
*4qkv*	3.00	Trimer (p) L	No	3	333					**0.38/0.82**
*4u5t*	3.30	Dimer (p) L	Yes	2	74					**0.34/0.81‡**
*6gbr*	3.14	Tetramer (p) L	No	4	284					**0.38/0.81‡**

† tNCS detected.

‡ Solutions obtained using only the 25 top-scoring *Rosetta* models for clustering and truncation.
